# Protective Effect of Curcumin on D-Galactose-Induced Senescence and Oxidative Stress in LLC-PK1 and HK-2 Cells

**DOI:** 10.3390/antiox13040415

**Published:** 2024-03-29

**Authors:** Semiramis Stephania García-Trejo, Tania Gómez-Sierra, Dianelena Eugenio-Pérez, Omar Noel Medina-Campos, José Pedraza-Chaverri

**Affiliations:** Department of Biology, Faculty of Chemistry, National Autonomous University of Mexico (UNAM), Mexico City 04510, Mexico; semi@ciencias.unam.mx (S.S.G.-T.); taniags@quimica.unam.mx (T.G.-S.); diane.eug@gmail.com (D.E.-P.); omarnoel@quimica.unam.mx (O.N.M.-C.)

**Keywords:** D-galactose, senescence, reactive oxygen species, oxidative stress, curcumin

## Abstract

D-galactose has been widely used as an inducer of cellular senescence and pathophysiological processes related to aging because it induces oxidative stress. On the other hand, the consumption of antioxidants such as curcumin can be an effective strategy to prevent phenotypes related to the enhanced production of reactive oxygen species (ROS), such as aging and senescence. This study aimed to evaluate the potential protective effect of curcumin on senescence and oxidative stress and endoplasmic reticulum stress induced by D-galactose treatment in Lilly Laboratories Culture-Porcine Kidney 1 (LLC-PK1) and human kidney 2 (HK-2) proximal tubule cell lines from pig and human, respectively. For senescence induction, cells were treated with 300 mM D-galactose for 120 h and, to evaluate the protective effect of the antioxidant, cells were treated with 5 µM curcumin for 24 h and subsequently treated with curcumin + D-galactose for 120 h. In LLC-PK1 cells, curcumin treatment decreased by 20% the number of cells positive for senescence-associated (SA)-β-D-galactosidase staining and by 25% the expression of 8-hydroxy-2′-deoxyguanosine (8-OHdG) and increased by 40% lamin B1 expression. In HK-2 cells, curcumin treatment increased by 60% the expression of proliferating cell nuclear antigen (PCNA, 50% Klotho levels, and 175% catalase activity. In both cell lines, this antioxidant decreased the production of ROS (20% decrease for LLC-PK1 and 10 to 20% for HK-2). These data suggest that curcumin treatment has a moderate protective effect on D-galactose-induced senescence in LLC-PK1 and HK-2 cells.

## 1. Introduction

Aging and senescence are closely related processes, although they are not synonymous. Aging is a natural and gradual process that reduces the function and maintenance of cellular systems that support their survival, increasing the risk of cell death. Senescence is part of the aging process and is characterized by the stable and irreversible cell cycle arrest caused by intracellular or extracellular stress or damage [1,2,3,4]. The goal of senescence is to limit the proliferation of damaged cells. However, chronic accumulation of senescent cells creates a proinflammatory environment that favors the incidence and progression of various age-related diseases [1,5].

For the initial phase of senescence, which is reversible, p53 and p21 proteins are essential for halting cell division, although cells remain metabolically active [6]. As the cells enter the second, irreversible phase, known as the maintenance phase, senescence becomes irreversible. Controlled by p16^INK4a^/pRb, this second phase results in the alteration of proteostasis, mitochondrial metabolism, and energy production [1,6]. Reactive oxygen species (ROS) and oxidative stress are crucial in both senescence phases since they modify the catalytic activity and conformation of proteins and alter protein–protein and protein–deoxyribonucleic acid (DNA) interactions and affect different specific signaling mechanisms. These events could favor cell cycle arrest and the senescent characteristic secretion of proinflammatory cytokines, chemokines, growth factors, matrix metalloproteinases, and insoluble protein components of the extracellular matrix [7,8,9,10,11,12,13].

It should be noted that there is a positive feedback loop between oxidative stress and DNA damage [14,15] since this structural alteration activates DNA repair systems, favoring cell cycle arrest [7,16], the accumulation of senescent cells, and the production of ROS [14], processes that are related to changes in antioxidants such as vitamins C and E, glutathione [17], catalase (CAT), glutathione peroxidase and superoxide dismutase (SOD) [4,15,18,19,20].

Oxidative stress also alters the protein folding in the endoplasmic reticulum (ER) causing an accumulation of misfolded proteins in the lumen of this organelle, a condition known as ER stress, that is counteracted by the activation of the unfolded protein response (UPR) to restore homeostasis of the ER through correct protein refolding and is suggested that both senescence and UPR are interconnected processes in which oxidative stress plays an important role [21].

In vitro senescence can be induced by radiation (ionizing and ultraviolet), antineoplastic drugs, activation of oncogenes, oxidative stress, and mitochondrial dysfunction [5]. One method used to study senescence in vitro is to expose cell lines to an exogenous factor [6], such as D-galactose, which is naturally found in the body, in dairy products, beets, plums, cherries, figs, and celery [22,23]. D-galactose has been widely used to induce cellular senescence and pathophysiological processes as ER stress [22,23,24,25,26,27,28,29,30,31,32] related to aging because it induces oxidative stress. However, it has been reported that different concentrations of D-galactose and exposure times are needed to induce senescence [24,25,26,27,28,33] and a different susceptibility and response of the same cell type in different species has also been observed [34,35,36].

It has been reported that under conditions of excess D-galactose, it is reduced by galactose reductase to galactitol, resulting in osmotic stress and mitochondrial dysfunction with the consequent increase in ROS generation. D-galactose can also be oxidized by galactose oxidase, generating hydrogen peroxide (H_2_O_2_) that alters redox homeostasis and causes oxidative stress, inflammation, mitochondrial dysfunction, and apoptosis [22,37].

As previously mentioned, oxidative stress is a causal factor in aging and cellular senescence; therefore, antioxidants have been used to delay these processes [4,20]. Curcumin [1,7-bis(4-hydroxy-3-methoxyphenyl)-1,6-heptadiene-3,5-dione] is a polyphenol extracted from the rhizomes of *Curcuma longa* L., a native Asian plant, that has a variety of traditional uses [38]. Curcumin has been reported to exert antioxidant, anti-inflammatory, antimutagenic, antimicrobial, hypoglycemic, and anticancer effects [39,40,41,42]. Moreover, its safety, tolerability, and non-toxicity have been reported in clinical trials [39]. Regarding its antioxidant effects, curcumin is a bifunctional antioxidant. It has two phenol groups which confer a high hydrogen-donating activity and directly scavenges ROS such as superoxide anion (O_2_^•−^), H_2_O_2_, and nitric oxide radicals [43]. Furthermore, this polyphenol increases the activity of antioxidant enzymes such as CAT, SOD, glutathione peroxidase, and heme oxygenase-1 [20,43,44] through the activation of nuclear factor erythroid 2-related factor (Nrf2) that induces the expression of genes coding for elements of the antioxidant system [43]. Furthermore, curcumin has also been considered an anti-aging and senolytic agent in different in vitro and in vivo models [7,20,45,46].

The present study aimed to evaluate the possible protective effect of curcumin on D-galactose-induced senescence in pig (Lilly Laboratories Culture-Porcine Kidney 1 cells, LLC-PK1) and human (Human Kidney 2 cells, HK-2) proximal tubule cell lines.

## 2. Materials and Methods

### 2.1. Cell Lines and Reagents

LLC-PK1 and HK-2 cells were obtained from the American Type Culture Collection (ATCC, Rockville, MD, USA). Bovine serum albumin (BSA), ethylenediaminetetraacetic acid (EDTA), citric acid, nitroblue tetrazolium (NBT), Nonidet p-40 (NP-40), D-galactose (cat. G5388), magnesium chloride, protease inhibitors, dihydroethidium (DHE), sodium deoxycholate, sodium dodecyl sulfate (SDS), ammonium molybdate, dimethyl sulfoxide, sodium fluoride, sodium pyrophosphate, sodium orthovanadate, trypan blue, Triton X-100, Tween 20, and the antibody against voltage-dependent anion channel (VDAC, cat. 065M4753V) were acquired from Sigma-Aldrich Co. (St. Louis, MO, USA). Curcumin of a high purity (≥98%, ALX-350-028-M050) was obtained from Enzo Life Sciences, Inc. (Ann Arbor, MI, USA). Dipotassium phosphate, ethylenediaminetetraacetic acid salt, potassium chloride, potassium biphosphate, sodium chloride, sodium bicarbonate, potassium ferrocyanide, and dibasic sodium phosphate were acquired from J.T. Baker (Ciudad de México, Edo. Mex, Mexico). Bradford’s reagent was acquired from Bio-Rad (Hercules, CA, USA), the X-Gal (cat. X4281C) was acquired from GoldBio Gold Biotechnology^®^ (St. Louis, MO, USA), and bisbenzimide H-33342 trihydrochloride (Hoechst) was acquired from Fluka^TM^ Chemicals (Buchs, Switzerland). Dulbecco’s Modified Eagle’s Medium high glucose (DMEM), fetal bovine serum (FBS), and penicillin/streptomycin were from Biowest (Riverside, MO, USA). The TrypLE Express solution was acquired from Thermo Fisher Scientific (Waltham, MA, USA). 2′,7′-Dichlorofluorescin diacetate (DCFH-DA) was from Molecular Probes (Eugene, OR, USA). Antibodies against glyceraldehyde 3-phosphate dehydrogenase (GAPDH, cat. AB9485), 8-hydroxy-2′-deoxyguanosine (8-OHdG, cat. AB6262), and proliferating cell nuclear antigen (PCNA, cat. AB2426) were acquired from Abcam (Boston, MA, USA). The antibody against mitofusin 2 (Mfn2, cat. 9482) was purchased from Cell Signaling Technology (Danvers, MA, USA). The antibody against kidney injury molecule-1 (KIM-1, cat. AF3689) was acquired from R&D Systems (Minneapolis, MN, USA). The antibodies against activating transcription factor 4 (ATF4, cat. SC-200), Klotho (cat. SC515940), and lamin B1 (cat. SC20682) were purchased from Santa Cruz Biotechnology Inc. (Dallas, TX, USA). Alexa Fluor^®^ 488 and Alexa Fluor^®^ 594 secondary antibodies were purchased from Jackson ImmunoResearch Inc. (West Grove, PA, USA). Anti-rabbit IRDye^®^ 800CW (cat. 926-32213) and anti-mouse IRDye^®^ 680RD (cat. 926-68072) secondary antibodies were from LI-COR Biosciences (Lincoln, NE, USA). All the other chemicals and reagents used were of analytical grade and were commercially available.

### 2.2. Cell Culture

LLC-PK1 and HK-2 cells were cultured in a humidified 5% carbon dioxide (CO_2_) atmosphere at 37 °C in DMEM cell culture medium supplemented with 10% FBS, 0.33% sodium bicarbonate (NaHCO_3_), and 1% penicillin/streptomycin. Cells were subcultured with TrypLE Express solution upon reaching 90% confluency. Experiments were performed with DMEM cell culture medium supplemented with 1% FBS and 1% penicillin/streptomycin.

### 2.3. Experimental Design

#### 2.3.1. First Stage (ROS Levels)

LLC-PK1 and HK-2 cells were treated with D-galactose (200, 300, and 500 mM) for 24 h to determine ROS production.

#### 2.3.2. Second Stage (Oxidative Stress/Senescence)

The following 4 experimental groups were established:

(1) Control: without treatment
(2) Curcumin: 5 μM curcumin for 144 h
(3) D-Galactose: 300 mM D-galactose for 120 h
(4) Curcumin + D-galactose: 24 h before D-galactose exposure, cells were treated with 5 µM curcumin and subsequently simultaneously treated with 5 µM curcumin + 300 mM D-galactose for 120 h. The cell culture medium was changed every 24 h in all experimental groups.

LLC-PK1 and HK-2 cells were treated with 300 mM D-galactose for 120 h to induce senescence. This scheme was selected based on first-stage ROS results, no evidence of senescence after 48 h of exposure to 500 mM D-galactose, and senescence data from other studies [27].

**Figure 1:**
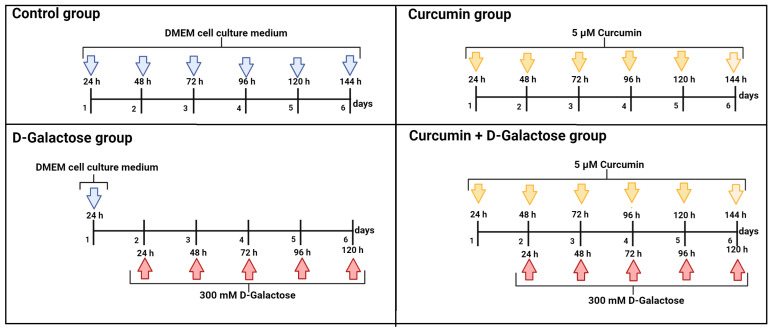
Scheme of treatment in the second stage of the experimental design in LLC-PK1 and HK-2 cells to evaluate the effects of curcumin on D-galactose-induced senescence. Control group: without treatment. Curcumin group: 5 μM curcumin for 144 h. D-galactose group: 300 mM D-galactose for 120 h and curcumin + D-galactose group: 24 h before D-galactose exposure, cells were treated with 5 µM curcumin and subsequently simultaneously treated with 5 µM curcumin + 300 mM D-galactose for 120 h. The cell culture medium was changed every 24 h in all experimental groups. Figure was created with Biorender.com (accessed on 23 March 2024).

The culture medium with D-galactose was changed every 24 h because the half-life of D-galactose is around 20 h [47]. To evaluate the protective effect of curcumin in this experimental model, 24 h before D-galactose exposure, cells were treated with 5 µM curcumin and subsequently simultaneously treated with 5 µM curcumin + 300 mM D-galactose for 120 h (Figure 1). The previous exposure of curcumin in cells is in order to stimulate cells to distribute biological resources that help to defend against a wide range of cellular stressors, such as D-galactose, and this adaptive mechanism could further reduce the damage more effectively than the response induced only by exposure to the stressor [39,48,49]. Furthermore, as in the D-galactose-treated group, the culture medium containing both curcumin and D-galactose was changed every 24 h. In the next sections, we will refer to this treatment scheme as curcumin + D-galactose treatment. Finally, a group treated only with 5 µM curcumin for 144 h was included in this experimental stage.

### 2.4. Oxidative Stress

#### 2.4.1. ROS Production

In a 96-well microplate, 64,000 cells/cm^2^ were seeded and allowed to adhere for 24 h in DMEM cell culture medium with 1% FBS. Subsequently, after the respective treatments, the culture medium was removed, and cells were washed three times with phosphate-buffered saline (PBS) pH 7. Following this, cells were incubated with 10 μM of DCFH-DA and 40 μM of DHE for 30 min in the dark at 37 °C [50,51]. Images and fluorescence intensity were visualized and determined in a Cytation 5 cell imaging multimode reader (Biotek Instruments, Inc., Winooski, VT, USA) at an excitation wavelength of 485/20 and 480/20 nm and emission of 528/20 and 576/20 nm for the dichlorofluorescein (DCF) and ethidium probes, respectively. The results were expressed as a percentage of change compared to the control group.

#### 2.4.2. Antioxidant Enzymes Activity

Cells were seeded in 60 mm Petri dishes and at the end of treatments, the medium was removed, and cells were rinsed with PBS three times. Half a milliliter of TryPLE express solution was added to dishes and after 3 min at 37 °C the suspension of cells was transferred to 1.5 mL centrifuge tube and centrifuged at 10,000× *g* at 4 °C for 20 min, the supernatant was discarded, and the pellet was sonicated thrice for 10 s each on ice. The homogenate was stored at −70 °C until use.

##### CAT Activity

It was determined by the method described by Hadwan and Abed [52]. Briefly, each cell extract was incubated with 20 mM H_2_O_2_ for 3 min, then 16.2 mM ammonium molybdate was added, and the absorbance at 374 nm was determined. The first-order reaction constant (*k*) was used as the unit of CAT enzyme activity. Results are expressed as *k*/mg protein.

##### SOD Activity

It was assayed according to Oberley and Spitz [53]. Briefly, each cell extract was mixed with 0.122 mM xanthine, 30.6 M NBT, 0.122 mM EDTA, 49 mM sodium carbonate (Na_2_CO_3_), and 0.001 units of xanthine oxidase and then the absorbance at 560 nm was determined. Results are expressed as units/mg protein, where one unit is the amount of SOD required to inhibit 50% of the NBT reduction. Protein content was determined using the Lowry method [54].

### 2.5. Evaluation of Cellular Senescence

#### 2.5.1. Nuclear Size

Hoechst (5 μg/mL) was added for 15 min to cells to counterstain nuclei, and then the images were viewed and analyzed on a Cytation 5-cell imaging multimode reader (Biotek Instruments, Inc.). Briefly, two independent experiments were performed (two biological replicates), and each independent experiment had three replicates of each group. The nuclear size was determined by measuring the nuclear area from at least 50 cells in 3 random fields per biological sample and using the Biotek Gen5 software 3.04. The procedure was made by two different analysts.

#### 2.5.2. β-Galactosidase Activity

β-galactosidase activity is detectable at pH 6.0 in senescent cells due to increased lysosomal content and decreased pH and is therefore a widely used marker of senescence [55,56]. For the senescence-associated (SA)-β-galactosidase assay, 62,000 cells/cm^2^ were seeded in a 12-well microplate and allowed to adhere for 24 h in a DMEM cell culture medium with 1% FBS. Subsequently, the treatments were carried out, and then DMEM cell culture medium was removed. Later, cells were fixed with 3% formaldehyde for 5 min at room temperature. After that, the cells were washed with PBS pH 6.0 and a solution of 1 mg/mL chloro-3-indolyl-beta-galactopyranoside (X-gal) was added, followed by incubation at 37 °C for 13 h [57]. Then, the X-gal solution was removed from the cells and washed two times with PBS pH 6.0. Hoechst was added to counterstain nuclei. Images were viewed and analyzed on a Cytation 5 cell imaging multimode reader (Biotek Instruments, Inc.).

#### 2.5.3. Senescence and Oxidative Stress-Associated Markers Evaluation by Immunocytochemistry

A sterile round coverslip was placed in each well of 12-well microplate, and 40,000 cells/cm^2^ were seeded and allowed to adhere for 24 h in DMEM cell culture medium with 1% FBS. Subsequently, treatments were carried out, and then the cells were fixed at room temperature with cold acetone for 15 min under agitation. Cells were permeabilized with 0.5% Triton X-100 in PBS for 15 min and then blocked with 2% BSA [58]. Antibodies against 8-OHdG (1:500), lamin B1 (1:1000), PCNA (1:1000), and Klotho (1:1000) were added and incubated overnight at 4 °C. Cells were then incubated for 2 h with secondary antibodies Alexa Fluor^TM^ 488 and Alexa Fluor^TM^ 594 at room temperature in the dark. Hoechst was added to counterstain nuclei. Images and fluorescence intensity were visualized and determined on the Cytation 5 cell imaging multimode reader (Biotek Instruments, Inc.).

#### 2.5.4. Senescence-Associated Markers Evaluation by Western Blot

LLC-PK1 and HK-2 cells were detached from 60 mM Petri dishes as described in Section 2.4.2. Lysis was performed with ultrasound using radioimmunoprecipitation assay (RIPA) buffer (50 mM Tris-HCl, pH 7.4, 150 mM NaCl, 1 mM EDTA, 0.5% sodium deoxycholate, 1% NP-40, 0.1% SDS, 25 mM sodium fluoride (NaF), 1 mM sodium pyrophosphate (Na_4_P_2_O_7_), 1 mM sodium orthovanadate (Na_3_VO_4_), 0.5 mM glycerophosphate-protease inhibitor cocktail). Lysates were centrifuged at 12,000× *g* for 20 min at 4 °C and the supernatant was collected and stored at −70 °C until use. Total protein content was determined by the Bradford assay [59].

Proteins were separated by SDS-polyacrylamide gel electrophoresis (PAGE), transferred to polyvinylidene fluoride membranes, and blocked for one hour with 5% skimmed milk. Membranes were incubated overnight at 4 °C with primary antibodies against ATF4 (1:1000), GAPDH (1:5000), KIM-1 (1:500), Mfn2 (1:500), PCNA (1:500) or VDAC (1:1000). Subsequently, membranes were washed and incubated with the appropriate secondary antibodies for 2 h. Proteins of interest were detected with the Odyssey Sa infrared imaging system (LI-COR, Inc., Lincoln, NE, USA). Image analysis was performed with Image Studio Lite software version 5.2 (LI-COR, Inc.).

### 2.6. Statistical Analysis

Data analysis was performed using GraphPad Prism 8.0 software (Boston, MA, USA, www.graphpad.com (accessed on 20 November 2023)). The data were tested for normality and analyzed by one-way analysis of variance (ANOVA) or Kruskal–Wallis’s test, followed by Tukey’s or Dunn’s multiple comparison tests, respectively. A *p* < 0.05 was considered significant. Results are expressed as mean ± standard error of the mean (SEM).

## 3. Results

### 3.1. First Stage (ROS Levels)

#### D-Galactose Enhanced ROS Production in LLC-PK1 and HK-2 Cells

**Figure 2:**
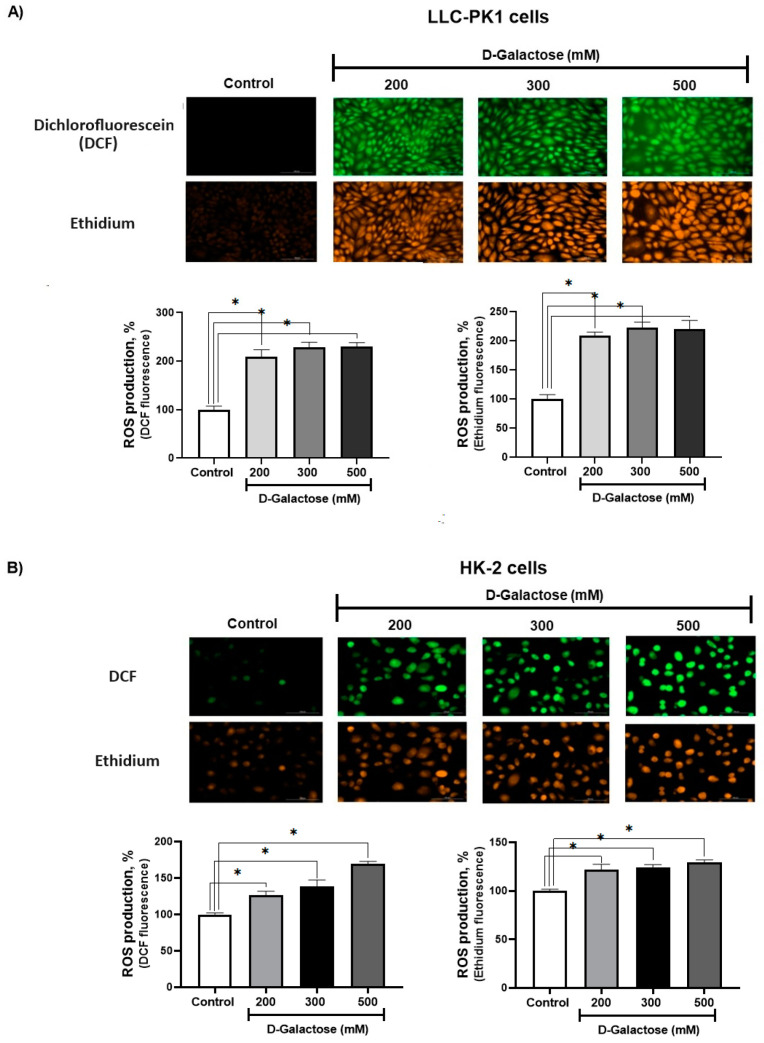
D-galactose induces reactive oxygen production (ROS) production in (**A**) LLC-PK1 and (**B**) HK-2 cells after 24 h of treatment. Data are shown as mean ± SEM, *n* = 3. * *p* < 0.05 vs. control. DCF = dichlorofluorescein. Objective: 20×, scale bar: 100 μM.

After 24 h of exposure, D-galactose increased ROS production in LLC-PK1 cells to ~200% with the three concentrations used (Figure 2A), while in HK-2 cells, ROS production increased from ~20% to 60% compared to the control group (Figure 2B).

### 3.2. Second Stage (Oxidative Stress/Senescence)

#### 3.2.1. Curcumin Treatment Decreased D-Galactose-Induced ROS Production in LLC-PK1 and HK-2 Cells

**Figure 3:**
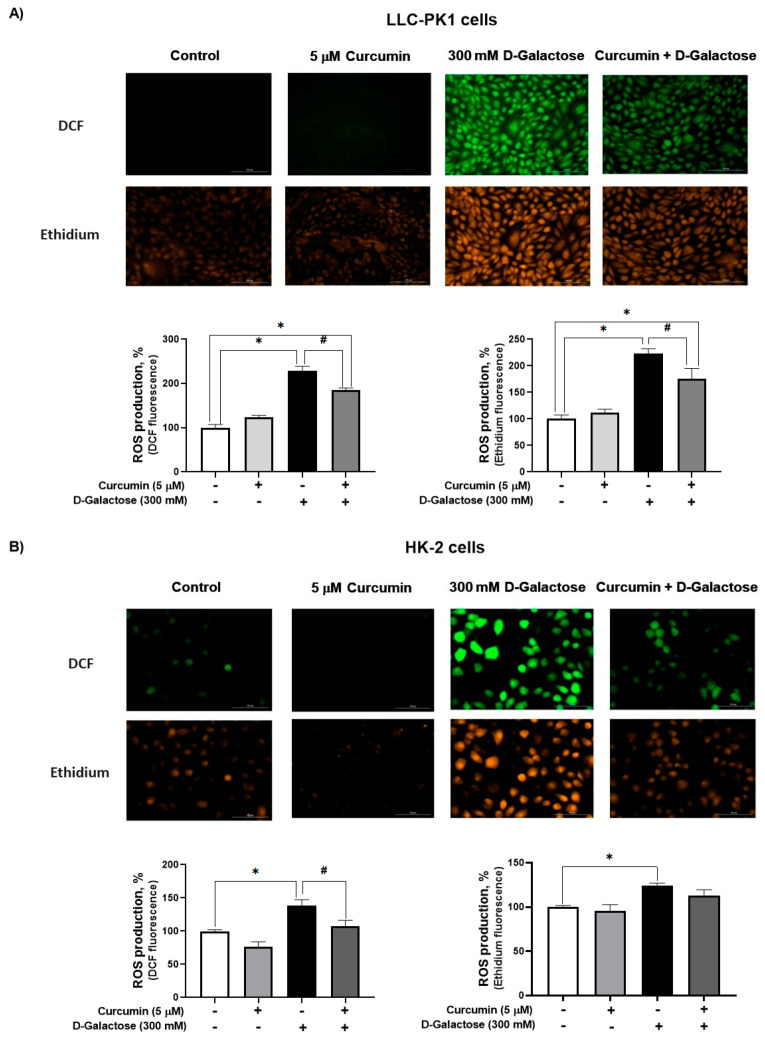
Curcumin decreases reactive oxygen production (ROS) production induced by 24 h exposure to D-galactose in (**A**) LLC-PK1 and (**B**) HK-2 cells. Data are shown as mean ± SEM, *n* = 3. * *p* < 0.05 vs. control (without curcumin and D-galactose), # *p* < 0.05 vs. D-galactose. DCF = dichlorofluorescein. Objective: 20×, scale bar: 100 μM.

Since DMEM with the corresponding treatment was changed every 24 h, ROS levels were assessed on the last day of treatment, i.e., at 120 h after initial exposure to D-galactose. It was observed that D-galactose increased ROS production by 30%, compared to the control group, and curcumin treatment decreased it by 20% in LLC-PK1 cells with both probes (Figure 3A). In HK-2 cells, D-galactose increases ROS production by 30%, compared to the control group, and curcumin treatment decreases it by 10 and 20% for DFC and ethidium, respectively (Figure 3B).

#### 3.2.2. Curcumin Treatment Prevented the D-Galactose-Induced Decrease in CAT Activity in HK-2 Cells

**Figure 4:**
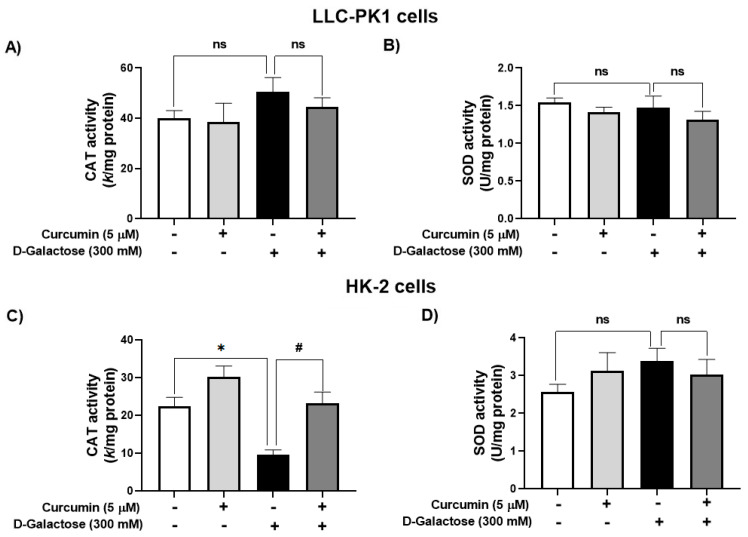
Activity of antioxidant enzymes in LLC-PK1 and HK-2 cells treated for 120 h with 300 mM D-galactose and treated with curcumin. (**A**) Catalase (CAT) and (**B**) superoxide dismutase (SOD) activities in LLC-PK1 cells. (**C**) CAT and (**D**) SOD activities in HK-2 cells. Data are shown as mean ± SEM, *n* = 3. * *p* < 0.05 vs. control (without curcumin and D-galactose), # *p* < 0.05 vs. D-galactose, ns: not significant.

D-galactose treatment did not modify the activity of CAT nor SOD in LLC-PK1 cells (Figure 4A,B). In contrast, in HK-2 cells, D-galactose decreased CAT activity by 57%, and curcumin treatment prevented this reduction (Figure 4C) but there were no significant changes in SOD activity (Figure 4D).

#### 3.2.3. Curcumin Did Not Prevent the D-Galactose-Induced Increase Nuclear Size in LLC-PK1 and HK-2 Cells

**Figure 5:**
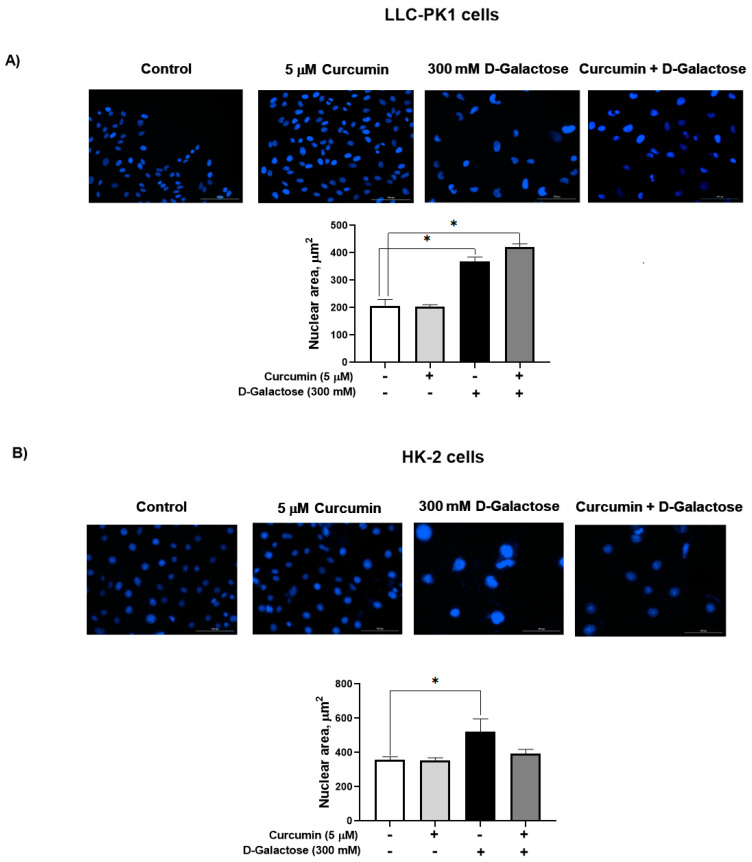
D-galactose exposure for 120 h increases nuclear area both in (**A**) LLC-PK1 and (**B**) HK-2 cells. Curcumin had no effect on the increase in nuclear size induced by D-galactose. Data are shown as mean ± SEM, *n* = 3. * *p* < 0.05 vs. control (without curcumin and D-galactose). Objective: 20×, scale bar: 100 μM.

D-galactose increased the nuclear size by 77% in LLC-PK1 cells (Figure 5A) and by 46% in HK-2 cells (Figure 5B). In both cell lines, curcumin had no effect on this senescence marker.

#### 3.2.4. Curcumin Prevent D-Galactose-Induced Changes in β-Galactosidase, PCNA and Lamin B1 in LLC-PK1 Cells

**Figure 6:**
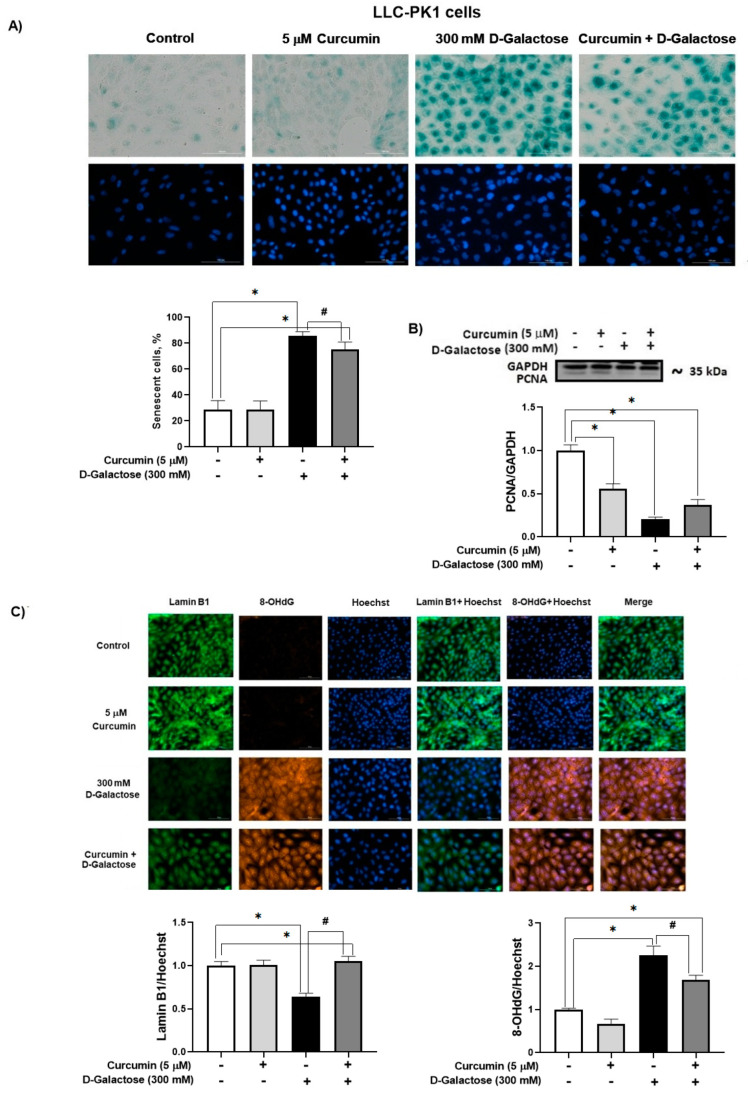
Curcumin prevents senescence induced by 120 h D-galactose exposure in LLC-PK1 cells. (**A**) Senescence-associated (SA)-β-galactosidase activity. (**B**) Proliferating cell nuclear antigen (PCNA) expression. (**C**) Lamin B1 and 8-hydroxy-2′-deoxyguanosine (8-OHdG levels). Data are shown as mean ± SEM, *n* = 3. * *p* < 0.05 vs. control (without curcumin and D-galactose), # *p* < 0.05 vs. D-galactose. GAPDH = glyceraldehyde 3-phosphate dehydrogenase. Objective: 20×, scale bar: 100 μM.

In the D-galactose-treated group, 86% of the cells were positive for SA-β-galactosidase staining (Figure 6A). Moreover, D-galactose-treated cells showed a decrease in PCNA (Figure 6B) and lamin B1 (Figure 6C) levels, and an increase in 8-OHdG (Figure 6C). Notably, curcumin treatment reduced by 20% SA-β-galactosidase positive cells (Figure 6A), prevented the loss of lamin B1 (40% increase), and decreased 8-OHdG by 25% (Figure 6C).

#### 3.2.5. Curcumin Prevented D-Galactose-Induced Decreases in PCNA and Klotho Levels in HK-2 Cells

**Figure 7:**
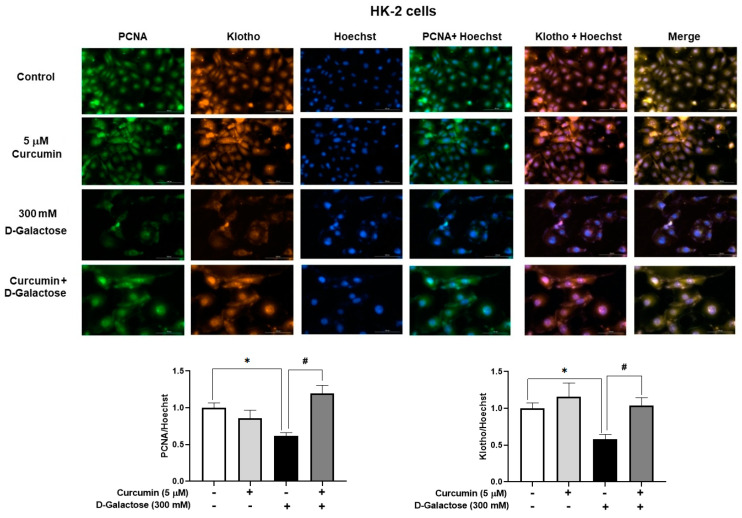
Curcumin prevents the loss of proliferating cell nuclear antigen (PCNA) and Klotho proteins induced by 120 h D-galactose treatment in HK-2 cells. Data are shown as mean ± SEM, *n* = 3. * *p* < 0.05 vs. control (without curcumin and D-galactose), # *p* < 0.05 vs. D-galactose. Objective: 20×, scale bar: 100 μM.

No positive SA-β-D-galactosidase staining was observed in HK-2 cells, so we decided to use other markers to assess cellular senescence. Treatment for 120 h with 300 mM D-galactose decreased the protein levels of PCNA and Klotho by 39% and 42%, respectively (Figure 7), while curcumin treatment prevented these changes (Figure 7).

#### 3.2.6. D-Galactose Treatment Did Not Induce Overexpression of KIM-1 in LLC-PK1 and HK-2 Cells

**Figure 8:**
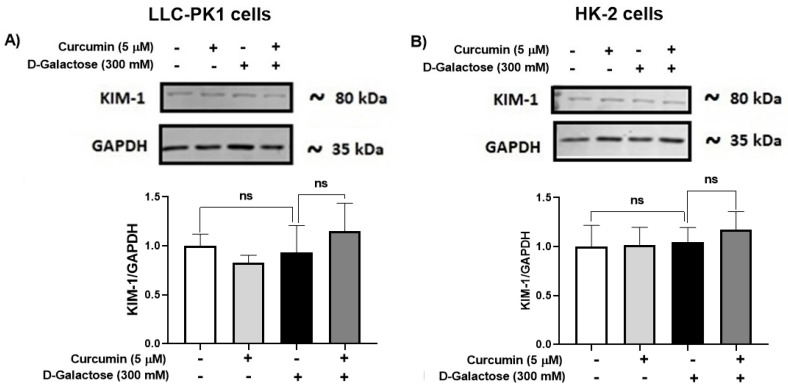
Kidney injury molecule-1 (KIM-1) expression in (**A**) LLC-PK1 and (**B**) HK-2 cells treated with D-galactose and curcumin. Data are shown as mean ± SEM, *n* = 3. ns: not significant.

Since senescence in renal cells is associated with tubular injury [60,61], KIM-1 expression was evaluated. However, the treatment for 120 h with D-galactose did not induce overexpression of KIM-1 in LLC-PK1 (Figure 8A) and HK-2 (Figure 8B) and it was also noted that curcumin did not modify the levels of this protein (Figure 8).

#### 3.2.7. D-Galactose Did Not Modify Expression of ATF4 and VDAC in LLC-PK1 and HK-2 Cells, and Decreased Mfn2 Expression in LLC-PK1 Cells and Curcumin Treatment Did Not Prevent It

**Figure 9:**
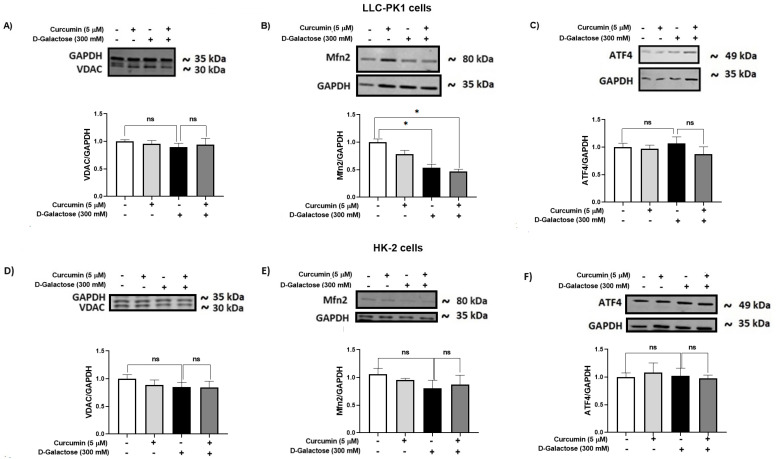
Curcumin effect on mitochondrial and ER stress-related proteins in LLC-PK1 and HK-2 cells exposed for 120 h to D-galactose. Qualitative and quantitative data of expression of (**A**) voltage-dependent anion channel (VDAC), (**B**) mitofusin 2 (Mfn2), and (**C**) ATF4 proteins in LLC-PK1 cells. The same type of data for expression of VDAC, Mfn2, and activating transcription factor 4 (ATF4) in HK-2 cells are shown in panels (**D**–**F**), respectively. Bars represent the average of 3 independent experiments ± SEM. * *p* < 0.05 vs. Control (without D-galactose and curcumin), ns: not significant.

It has been reported that the accumulation of senescent cells induces mitochondrial dysfunction and ER stress [62]. Thus, the expression of some proteins related to these organelles, such as VDAC (Figure 9A,D), Mfn2 (Figure 9B,E), and ATF4 (Figure 9C,F), were evaluated in LLC-PK1 (Figure 9A–C) and HK-2 (Figure 9D–F).

D-galactose treatment decreased Mfn2 levels in LLC-PK1 cells and curcumin treatment did not prevent it (Figure 9B). In HK-2 cells, D-galactose treatment seems to decrease Mfn2 expression (Figure 9E), although its effect was not significant. Moreover, levels of VDAC and ATF4 were not modified by D-galactose treatment in both cell lines (Figure 9A,C,D,F).

## 4. Discussion

D-galactose has been widely used in in vitro accelerated aging models with various concentrations and exposure times [24,25,26,27,28,29,33,63] and it has been established that one of the primary mechanisms by which this sugar exerts its senescent effect is through increased ROS production [22,37]. Noteworthy, 300 mM D-galactose increased ROS levels in both cell lines. When LLC-PK1 and HK-2 cells were exposed to 200, 300, and 500 mM D-galactose for 2 h an increase in ROS levels was observed and remained up to 24 h (Figure 2).

It was observed that D-galactose increased ROS production in both LLC-PK1 and HK-2 cells, an effect that was prevented by curcumin (curcumin + D-galactose group) (Figure 3). During the experimental protocol, the cell culture media with the corresponding treatments were changed every 24 h, so that the metabolism of D-galactose by galactose reductase and galactose oxidase was continuous, which caused the cells to be exposed to an oxidizing environment [22,37]. The increase in ROS had no effect on the activities of antioxidant enzymes as CAT and SOD in LLC-PK1 cells (Figure 4A,B) but a decrease in CAT activity (Figure 4C) was observed without changes in SOD activity in HK-2 cells (Figure 4D). Curcumin treatment did not cause changes in the activities of these enzymes in any of these cell lines. In addition, 8-OHdG levels also can be considered as an oxidative stress marker, as it is one of the predominant forms of free radical-induced oxidative damage to DNA structure. Similar to the results observed in ROS levels, D-galactose increased 8-OHdG levels and curcumin prevented this increase in LLC-PK1 cells (Figure 6C).

Senescent cells are characterized by enlarged and irregularly shaped nuclei, caused by cell cycle arrest in the G1/G2 phase, loss of lamin B1, changes in cytoskeletal elements as increased formation of actin fibers that accumulate in the cytoplasm and nuclei and forcing the cells to flatten, so an increased nuclear surface area is projected [1,7,16,64]. In our experimental model, an increase in nuclear size is also observed in senescent LLC-PK1 and HK-2 cells, and nuclear morphology has even been proposed as a biomarker of cellular senescence because it is common in some cells and species [16].

Noteworthy, in both cell lines, enlarged and irregular nuclei are observed and it has been indicated that cells with larger nuclear size do not replicate [8,13,64,65]. This finding is related to another unique phenotype of senescent cells, the formation of senescence-associated heterochromatin foci (SAHF), specialized regions of facultative heterochromatin that decrease the expression of proliferative senescence-promoting genes [66,67].

Although curcumin treatment did not attenuate the increase in nuclear size in LLC-PK1 or HK-2 cells, it prevented the decrease in PCNA in HK-2 cells (Figure 7), but not in LLC-PK1 cells (Figure 6B). Low levels of PCNA are associated with senescence because it is a protein that participates in the cell cycle. In some cell lines, it has been observed that the increase in PCNA is evident from 8 to 12 h after entry into the G1 phase, reaching a peak in the G1/S phase and decreasing during the S phase of the cell cycle [68]. Furthermore, PCNA acts as a cofactor of DNA polymerase delta and is associated with chromatin remodeling and DNA replication and repair processes [69,70,71]. In the group treated with D-galactose, the decrease in PCNA could affect DNA repair processes, which would partly explain the increase observed in 8-OHdG levels (Figure 6C), since, as previously mentioned, the metabolism of D-galactose generates ROS that damage DNA which can cause the loss of epigenetic information and be responsible for mutagenesis [8,72]. Curcumin, through its antioxidant properties, decreased DNA damage and reduced the number of senescent cells, preventing the decrease in lamin B1 [73] in LLC-PK1 cells (Figure 6C). It has been observed that the expression of lamin B1 is decreased by oxidative stress and is related to the senescent phenotype [74,75].

Furthermore, in our experimental model, in LLC-PK1 cells, in the group treated with D-galactose, there is an increase in the number of cells positive for SA-β-D-galactosidase staining, while in the curcumin-treated group, the number of positive cells decreases (Figure 6A), which could be associated with a lower number of senescent cells, which present an intralysosomal pH of 6.0 [8], and not in proliferating ones [13,16,55,56]. However, no positive SA-β-D-galactosidase staining was observed in HK-2 cells. Although this is one of the most used methods to detect senescence, it has the limitation that SA-β-D-galactosidase activity is not a completely specific marker of cellular senescence, and the evaluation of other markers is required [1,55,56].

On the other hand, in HK-2 cells treated with D-galactose, a decrease in Klotho protein levels was observed, which is restored with curcumin treatment (Figure 7). Klotho is a protein produced mainly in the kidneys, brain, pancreas, and other tissues [76] that inhibits four pathways that have been linked to aging, including transforming growth factor β (TGF-β), insulin-like growth factor 1 (IGF-1), Wingless-related integration site (Wnt), and nuclear factor kappa B (NF-κB), which could induce cellular senescence, apoptosis, inflammation, immune dysfunction, fibrosis, and neoplasia. It has been described that if Klotho decreases, an increase in the expression of proteins associated with senescence, such as p16, p21, and p53 is observed [63,77,78]. Furthermore, it has been observed that Klotho knockout mice have a shorter lifespan and that transgenic mice overexpressing Klotho have a longer lifespan than wild-type mice [79]. Therefore, the decrease in this protein after D-galactose treatment and the prevention of this change by curcumin agree with the induction and prevention of senescence by D-galactose and curcumin, respectively.

In addition, Klotho may have antioxidant activity by increasing CAT and SOD enzymes through Nrf2 and forkhead box O (FoxO) transcription factors [31,80]. Likewise, it has been shown that Klotho deficiency increases ROS production and enhances oxidative stress, while its overexpression can attenuate the production of O_2_^•−^ [63,81,82]. Therefore, in our experimental model, it is possible that Klotho participates in the antioxidant response of the cell. In HK-2 cells, it is observed that curcumin treatment restores CAT activity, probably through its bifunctional antioxidant properties [20,31,43,44] or by preventing the decrease in Klotho [77,78].

On the other hand, an increase in KIM-1 has been observed in some models of senescence [60,61], which is associated with tubular injury. In our experimental model, no change in the expression of this protein was observed. However, other more sensitive markers could be evaluated to rule out or prove that D-galactose induces tubular injury.

Furthermore, it has been described that the accumulation of senescent cells induces mitochondrial dysfunction and stress in the ER [83,84]. Senescence activates inositol 1,4,5-trisphosphate receptors (IP3R) to release calcium from the ER and causes the VDAC/MCU channels to initiate the flow towards the mitochondria, the increase in calcium levels in senescent cells decreases the membrane potential, induces alterations in the calcium transport and electron transport chain and therefore increases ROS production [83,85]. Moreover, intracellular calcium changes activate the PERK/eIF2α/ATF4 pathway that is related to the regulation of a variety of genes involved in proliferation, differentiation, metastasis, autophagy, and antioxidant response. Increased expression of ATF4 has been observed to facilitate the progression of age-related diseases [28]. In our experimental model, no changes are observed in the expression of VDAC and ATF4. Regarding Mfn2, our results show a decrease in the levels of this protein when LLC-PK1 cells were exposed to D-galactose. This could be indicative of an alteration in the mitochondria and the ER since Mfn2 establishes communication between these two organelles. Interestingly, Mfn2 has also been described to have several functions in various cellular processes such as proliferation and cell death [86,87,88], so its role in senescence must be studied in detail.

**Figure 10:**
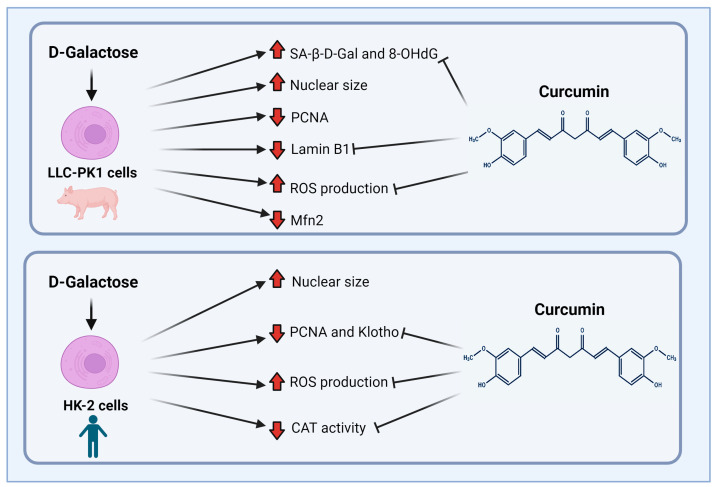
Scheme of the main findings of D-galactose treatment and curcumin treatment in LLC-PK1 and HK-2 cells. Abbreviations: CAT: catalase; Mfn2: mitofusin 2; 8-OHdG: 8-hydroxy-2′-deoxyguanosine; PCNA: proliferating cell nuclear antigen; ROS: reactive oxygen species; SA-β-gal: senescence-associated beta-galactosidase. Figure was created with Biorender.com (accessed on 1 February 2024).

Finally, although both cell lines (LLC-PK1 and HK-2) are proximal tubule cells, it is interesting how the response to the same stimulus changes depending on the studied species, (pig vs. human). Figure 10 shows the main findings of this study. LLC-PK1 cells seem to be more susceptible than HK-2 cells to damage induced by D-galactose. This susceptibility and the differential response of the species of the same cellular line had already been observed with some nephrotoxic agents [34,89], so it is important to know and understand the associated mechanisms to establish models that contribute to explaining the mechanisms involved in the senescent phenotype.

## 5. Conclusions

Our results show that curcumin prevents changes induced by D-galactose in different senescence markers, notably β-galactosidase activity (decrease of 20% in LLC-PK1 cells) and PCNA (decrease of 60% in HK-2 cells). Moreover, it decreases oxidative stress (ROS reduction of 20% in both cell lines) and oxidative damage (8-OHdG reduction of 25% in LLC-PK1 cells) caused by D-galactose treatment. Therefore, curcumin has a moderate protective effect on D-galactose-induced senescence and oxidative stress in LLC-PK1 and HK-2 cells and could be further considered as a possible therapeutic agent.

This study provides evidence of some of the proteins affected by treatment with D-galactose in this experimental model and the possible targets of curcumin, which could support future studies on the development of alternative therapeutic approaches for aging and its related diseases.

Finally, the present study also presents proof of the variability of the response of the species to the same stimulus, which can lead to new research aimed at understanding the mechanisms involved. Moreover, it underscores the significance of considering this fact when employing animal models of human diseases.

## Data Availability

The data that support the findings of this study are available from the corresponding author upon reasonable request.
